# A Timely Shift from Shotgun to Targeted Proteomics and How It Can Be Groundbreaking for Cancer Research

**DOI:** 10.3389/fonc.2017.00013

**Published:** 2017-02-20

**Authors:** Sara S. Faria, Carlos F. M. Morris, Adriano R. Silva, Micaella P. Fonseca, Patrice Forget, Mariana S. Castro, Wagner Fontes

**Affiliations:** ^1^Mastology Program, Federal University of Uberlandia (UFU), Uberlandia, Brazil; ^2^Laboratory of Biochemistry and Protein Chemistry, Department of Cell Biology, Institute of Biology, University of Brasilia, Brasília, Brazil; ^3^Department of Biochemistry and Molecular Biology, University of Southern Denmark, Odense, Denmark; ^4^Department of Anesthesiology and Perioperative Medicine, Universitair Ziekenhuis Brussel, Vrije Universiteit of Brussel, Brussels, Belgium

**Keywords:** targeted proteomics, cancer, biomarkers, mass spectrometry, diagnosis

## Abstract

The fact that cancer is a leading cause of death all around the world has naturally sparked major efforts in the pursuit of novel and more efficient biomarkers that could better serve as diagnostic tools, prognostic predictors, or therapeutical targets in the battle against this type of disease. Mass spectrometry-based proteomics has proven itself as a robust and logical alternative to the immuno-based methods that once dominated the field. Nevertheless, intrinsic limitations of classic proteomic approaches such as the natural gap between shotgun discovery-based methods and clinically applicable results have called for the implementation of more direct, hypothesis-based studies such as those made available through targeted approaches, that might be able to streamline biomarker discovery and validation as a means to increase survivability of affected patients. In fact, the paradigm shifting potential of modern targeted proteomics applied to cancer research can be demonstrated by the large number of advancements and increasing examples of new and more useful biomarkers found during the course of this review in different aspects of cancer research. Out of the many studies dedicated to cancer biomarker discovery, we were able to devise some clear trends, such as the fact that breast cancer is the most common type of tumor studied and that most of the research for any given type of cancer is focused on the discovery diagnostic biomarkers, with the exception of those that rely on samples other than plasma and serum, which are generally aimed toward prognostic markers. Interestingly, the most common type of targeted approach is based on stable isotope dilution-selected reaction monitoring protocols for quantification of the target molecules. Overall, this reinforces that notion that targeted proteomics has already started to fulfill its role as a groundbreaking strategy that may enable researchers to catapult the number of viable, effective, and validated biomarkers in cancer clinical practice.

## Cancer and How Proteomic Tools Can be of Help

Cancer remains a major cause of mortality worldwide, despite continuous progress in detection, diagnosis, and therapy of these maladies (1–3). According to the World Health Organization, more than 14.1 million new cancer cases and 8.2 million cancer-associated deaths were reported globally in 2012, while it is estimated that we will see an increase of over 70% new cases during the next two decades. Lung tumors remain the most common type of cancer in the world, both in terms of new cases and fatalities while breast cancer is the second most common cancer overall, but ranks fifth as cause of death due to the relatively favorable prognosis; these are followed, as related to incidence, by colorectal cancer (CRC) and prostate cancer (4). Early diagnosis improves the likelihood of successful treatment and can be lifesaving. The obvious epidemiological relevance of cancer has led to the publication of many studies, but only about 30 molecular tumor biomarkers are currently recommended in clinical practice guidelines across all cancer types, which serves as a statement to just how urgent it is to find more and better cancer biomarkers (5–7).

Diagnostic biomarkers are highly important for early detection and diagnosis of cancer, making their discovery an urgent requirement for improving outcomes. The complexity and heterogeneity of cancer makes it clear that the disease evolves *via* multiple pathways and it is actually the result of a combination of tumorigenesis, tumor relapse, and metastasis, involving complex protein networks and clinical events. Since malignant transformation that culminates in cancerous cells involves changes in protein expression, posttranslational modifications, and degradation, all of which in turn influence the molecular circuitry in these cells, it stands to reason that protein analysis is a good way of identifying modifications and interactions through which the disease evolves (4).

Proteins are ubiquitous molecules involved in virtually every single biological phenomena, from providing cell structure to closely regulating host responses to infection and inflammation (8). One can then assume that unraveling the many interactions between these molecules is paramount to better understand and intervene in any disease process, including cancer. Proteomic studies are needed to cope with such a daunting task, since the mere extrapolation of genomic data has proved to be insufficient in making sense of the real-world complexity of the interaction and function of biological processes regulated by proteins. This is because mechanisms such as allosteric regulation, posttranslational modifications, alternative splicing, and dynamic protein–protein interactions render genetic prediction tools incomplete, since they cannot accurately predict protein abundance or activity (9). Recent developments in pathway analysis provide ways to gather insights into the biology of the identified genes and proteins in cancer patients who can be associated with a phenotype by genomic analysis. Thus, new information can be obtained from global analyses of proteins. Integrated genomics and proteomics analyses provide a more well-rounded view of cancer biology and are able to generate better predictions for clinical phenotypes. The advent of new technologies to study the genome gave birth to several tools, including proteomics, that can better serve the purpose of understanding the biological processes behind cancer. In that sense, studies based on proteomic analysis help to systematically and simultaneously identify different proteins expressed in a given cell type or biological fluid, while enabling the monitoring of posttranslational modifications, structural changes, and other interactions involving proteins (10).

The adoption of proteomic approaches represents a shift in the general strategy to unravel the processes involved in cancer. For a long time, many laboratories around the world used the identification or quantification of specific protein species as predictors of the physiological states of cancer cells (11), primarily through immunological assays, such as ELISA, Western blot (WB), and other immunohistochemistry (IHC)-based tools to quantify individual proteins. However, these types of quantification methods are laborious and costly and often do not allow multiplexed assays nor represent the absolute abundance of the actual biomarkers in a biological sample (12, 13).

Proteomic tools, on the other hand, have greatly progressed over the years and have, in more than one instance, replaced the aforementioned methods in the study of cancer biomarkers. The first report of a proteomic analysis of cancer was based on a two-dimensional gel electrophoresis (2DGE) run that had selected spots identified by matrix-assisted laser desorption ionization mass spectrometry (MS). A total of 11 proteins were identified in lysates of human A375 melanoma cells (14). Gel-based proteomics has greatly contributed for significant development in cancer research, more specifically on the study of colorectal (15), pancreatic, and breast cancer (16, 17). Throughout the last decade, gel-based experiments have been partially replaced by MS-based techniques, due to some advantages of the latter. Nevertheless, 2DGE-based approaches are still used in cancer research (18) and have been shown to possess a few specific advantages, as recently published by Rogowska-Wrzesinska et al., despite it being a laborious and time-consuming method that presents lower throughput when compared to liquid chromatography-mass spectrometry (LC-MS)-based proteomics (19).

The design of new strategies for sample fractionation, labeling, processing, and analysis through bioinformatic tools, combined with the ever-increasing speed and sensitivity of the latest generation of mass spectrometers have propelled the proteomic field of study to a point in which it is feasible to identify, quantify, and monitor robust sets of data regarding protein biomarkers expression, posttranslational modifications, and other molecular aspects of cancer that may be clinically relevant. Being able to compare the ratios of protein abundance, posttranslational modifications, complex formation, and protein interaction networks, among disease states, generates a wide range of possibilities for studying the progression of cancer. These kind of studies have been of the utmost importance for the development of novel diagnostic and therapeutical tools, while also enabling a better understanding of the processes that occur during the progression of cancer (20).

There are many ways to go about conducting a proteomics-based investigation of cancer and recently a paradigm shift has been taking place, in which a clear maturing of the technology can easily be seen coexisting with changes in the dynamics of both discovery-based (i.e., shotgun proteomics) and hypothesis-based (i.e., targeted proteomics) methods. In the following pages, we not only dwell on the general aspects of these different proteomic approaches but also provide a wide range of examples on just how targeted proteomics has already provided inspiring results in the search of novel and useful biomarkers relevant to different aspects of cancer research. Out of the more than 100 studies reviewed in the present text, we were able to compile a comprehensive table that shows how different proteomic tools have already broken the surface of streamlined biomarker discovery through the use of a variety of samples in several types of cancer. An expanded and unabridged version of these findings can be seen in Tables 1 and 2, at the end of the present review. Some of these studies are more deeply discussed in the section dedicated to describing biomarker discovery in specific types of cancer.

**Table 1:** **A compilation of some of the most relevant studies that made use of samples from human cancer patients for the search of molecular biomarkers relevant to the diagnosis, prognosis, and/or therapeutical strategies in cancer research**.

Reference	Type of cancer	Methodology				Sample	Patients		Statistically validated targets		Type of biomarker
		Prefractioning/enrichment	Target selection	Number of targets	Mass spectrometry (MS) approach		Controls	Cancer	Protein ID	Number of proteins	
(21)	Bladder	–	Isobaric tags for relative and absolute quantitation (iTRAQ) shotgun proteomics	63	Stable isotope dilution-selected reaction monitoring (SID-SRM)	Urine	80	76	Adiponectin, Afamin, apolipoprotein A-II precursor, CERU, complement C4 gamma chain; prothrombin	6	Diagnostic

(22)	Breast	Affinity depletion of 14 abundant proteins	Label-free (LF) shotgun proteomics	34	SID-SRM	Plasma	4	–	–	–	Diagnostic

(23)	Breast	SCX	iTRAQ shotgun proteomics		SID-SRM and LF-SRM	Tissue	13	19	Decorin; endoplasmin; 40S ribosomal; alpha actin; 14-3-3 zeta-delta	49	Prognostic

(24)	Breast	Affinity depletion of abundant proteins	Transcriptomics; cell culture shotgun proteomics; bioinformatics	107	Labeled reference peptide (LRP)-SRM	Plasma	–	76	Fibronectin, clusterin, gelsolin and α-1-microglobulin/Inter-α-trypsin		Prognostic

(25)	Breast	Immunoprecipitation	LF shotgun proteomics	4	SID-SRM	Serum; Tissue	56	56	PDCD4, CGN, G3BP2, OCIAD1	4	Therapeutic

(26)	Breast	2D-LC	Cell culture shotgun proteomics; literature	319	SID-SRM	Tissue	–	244	CLTC, DPYSL2; ABAT; many others		Diagnostic; prognostic

(27)	Breast	Enrichment for membrane proteins	iTRAQ shotgun proteomics	49	SRM	Tissue	–	27	MFAP4; GP2	23	Prognostic

(28)	Breast	SCX; Fe-IMAC	iTRAQ shotgun proteomics	19	SID-SRM	Tissue	–	12	PDS5; T1208; S352; S417; S13	15	Prognostic

(29)	Breast	–	Transcriptomics database; LF shotgun proteomics;	20	SID-SRM	Tissue	–	96	KPNA2; CDK1	2	Prognostic

(30)	Breast	–	LF shotgun proteomics	3	LF-SRM	Plasma	204	216	ApoA1; hemopexin; angiotensin; preprotein	3	Diagnostic

(31)	Breast	Off-gel IEF	iTRAQ shotgun proteomics; western blotting	3	SID-SRM	Tissue	10	10	Cofilin-1; p23	2	Prognostic

(32)	Breast	–	Cell culture shotgun proteomics; literature	1	SID-SRM	Tissue	–	40	HER2		Diagnostic; prognostic

(33)	Breast	Precipitation and ultrafiltration	Literature	5	SID-SRM	Plasma	20	20	IGF1, IGF2, IBP2, IBP3, and A2GL	5	Diagnostic

(34)	Breast	–	LF shotgun proteomics	100	LF-pSRM	Tissue	–	51	ENPP1; UQCRFS1; GNB4	–	Therapeutic

(35)	Breast	SPE	Literature	6	SID-SRM	Serum	6	6	Hyp3-bradykinin; Des-Arg9-bradykinin; Fib-α; C4a; ITIH4; Bradykinin	6	Diagnostic

(36)	Breast	Affinity depletion of abundant proteins; immunoaffinity enrichment	Literature	1	SID-SRM	Serum	60	60	sTfR-transferrin receptor	1	Therapeutic

(37)	Breast	Membrane protein extraction	Literature	1	SID-SRM	Tissue	–	60	P-gp	1	Prognostic

(38)	Colon	SDS-PAGE	Literature	60	18O SID-SRM	Feces	5	5	L-plastin, filamin A, S100A9, hemoglobin, myeloperoxidase	5	Diagnostic

(15)	Colon	SDS-PAGE, RP, and SEC HPLC	LF shotgun proteomics	40	SID-SRM	Feces	7	8	a-1-antityrpsin, a-1-acid glycoprotein, complement C3, fibrinogen, haptoglobin, hemoglobin a, hemoglobin b, myeloblastin, transferrin	9	Diagnostic

(39)	Colon	Affinity depletion of 6 abundant proteins; SDS-PAGE	LF shotgun proteomics	8	LF-SRM	Plasma	48	48	CLU	1	Diagnostic

(40)	Colon	–	Literature	7	SID-SRM	Serum	259	172	ORM1, GSN, C9, HABP2 SAA2, C3	6	Diagnostic

(41)	Colon	Ultracentrifugation and filtration to select NOP	LF shotgun peptidomics	1	SID-SRM	Urine	25	24	Collagen type 1	1	Prognostic

(42)	Colon	Laser microdissection; SDS-PAGE	Literature	22	SID-SRM	Tissue	–	3	B-catenin; c-Src; c-Myc; PP2A	4	Prognostic

(43)	Colon	Cell membrane fractionation	iTRAQ shotgun proteomics	105	SID-SRM	Tissue	16	33	ITGA5, GPRC5A, PDGFRB, TFRC, Others (see Table 2)	44	Diagnostic; prognostic; therapeutic

(44)	Colon	–	Literature	3	Hyperplex-SRM	Tissue	–	37	AHCY, CTSD, LYZ	3	Prognostic

(45)	Endometrial	SCX	iTRAQ shotgun proteomics	1	mTRAQ-SRM	Tissue	1	1	PK	1	Diagnostic

(46)	Gastric	Laser microdissection	Literature	1	SID-SRM	Tissue	–	130	MET	1	Prognostic; therapeutic

(47)	Glioblastoma	–	Transcriptomics; shotgun proteomics; literature	100	SID-SRM	Tissue	–	–	Malectin; calnexin; LDHA; IDH; and many others	32	Therapeutic

(48)	Kidney	–	–	–	SWATH	Tissue	9	9	–	–	Diagnostic

(49)	Liver	Lectin enrichment for glycoproteins	Literature	10	SID-SRM	Plasma	30	10	AACT, A1AT, A1AG1, CERU	4	Diagnostic

(50)	Liver	Lectin enrichment for glycoproteins	Literature	2	SID-SRM	Plasma	3	3	A1AT; FETUA		Diagnostic

(51)	Liver	–	cDNA microarray; copy number variation; somatic mutation; epigenetic; quantitative proteomics data	50	LRP-SRM	Serum	36	36	ANLN; FLNB; C4A; AFP	4	Diagnostic

(52)	Liver	Affinity depletion of 6 abundant proteins	Literature	1	SID-SRM	Serum	95	60	AFP	1	Diagnostic

(53)	Liver	mHFER enrichment for glycopeptides	shotgun proteomics	49	iCCM-SRM	Serum	10	3		14	

(54)	Liver	Affinity depletion of 6 abundant proteins	LF shotgun proteomics	4	SID-SRM	Plasma	10	1	AGP; vitronectin; afamin precursor; Kininogen1 precursor	4	Diagnostic; prognostic; therapeutic

(55)	Liver	–	Literature	2	SID-SRM	Plasma	40	41	Vitronectin; AGP	2	Diagnostic

(56)	Liver	Aptamer-based fractionation	Gel-based proteomics	1	SID-SRM	Serum	24	26	ApoA1	1	Diagnostic; prognostic

(57)	Liver	Aptamer-based proteominer depletion of abundant proteins	2D-DIGE	43	SID-SRM	Serum	6	6	AFP, AFP-L3, DCP	–	Diagnostic

(58)	Liver	SDS-PAGE	LF shotgun proteomics	31	SID-SRM	Tissue	–	50	DDX39; FBLN5; MARCKS; SERPINH1; VCAN	5	Diagnostic

(59)	Liver	Hp affinity isolation	Literature	1	SID-SRM	Plasma	20	10	Haptoglobin-T3	1	Prognostic

(60)	Liver	–	Literature	2	O18-SID-SRM	Serum	10	10	CLU-1; VIT-2	2	Prognostic

(61)	Lung	Lectin fractionation	Literature	2	SID-SRM	Plasma	33	33	AGP, CP	2	Prognostic

(62)	Lung	Affinity depletion of 6 abundant proteins	LF shotgun proteomics	34	SID-SRM	Pleural effusion	86	39	ALCAM, CDH1, MUC1, SPINT1, THBS4	5	Diagnostic

(63)	Lung	Enrichment for desthiobiotinylated and tyrosine-phosphorylated peptides	LF shotgun proteomics	264	LRP-SRM	Tissue	5	5	Kinases		Therapeutic

(64)	Lung	Microdissection	Literature	1	SID-SRM	Tissue	–	23	EGFR	1	Therapeutic

(65)	Lung	Affinity depletion of albumin and IgG	Literature	95	SID-SRM	Plasma	72	30	ACTN1; ALDOA; ENO1; FLNA; G6PD; GPI; HSP90B1; ICAM1; ILK; LDHB; MSN; PGK1; PKM2; SPP1; TALDO1; THBS1; ZYX	17	Diagnostic

(66)	Lung	Affinity depletion of albumin and IgG	Literature	371	SID-SRM	Plasma	123	124	ISLR; BGH3; FIBA; TSP1; TETN; COIA1; LG3BP; LRP1; FRIL; PRDX1; GRP78; ALDOA; GSLG1	13	Diagnostic

(67)	Lung	Immunoaffinity	Databases, literature	5	SID-SRM	Serum	12	12	TIMP1, SLPI, TFPI, TFPI2, and CEA	5	Diagnostic

(68)	Lung	Laser microdissection	LF shotgun proteomics	6	In sample internal standard-SRM	Tissue	–	27	Napsin-A, hAG-2	2	Prognostic

(69)	Lung	–	LF shotgun proteomics	705	LF-SWATH	BALF	12	12	Haptoglobin, complement C4-A, glutathione *S*-transferase	44	Diagnostic

(70)	Lung	–	Literature	1	SID-SRM	Serum	50	160	Hp (subunits α and β)	1	Diagnostic

(71)	Lung	–	Literature	2	SID-SRM	Serum	99	100	SAA1 and SAA2	2	Diagnostic

(72)	Lung	Immunoaffinity	Literature	3	SID-SRM	Serum	4	6	ProGRP isoform 1, ProGRP isoform 3, NSE	3	Diagnostic

(73)	Lung	SDS-PAGE	LF shotgun proteomics	10	SID, LRP, and LF-SRM	Tissue	–	–	β-galactosidase; alkaline phosphatase	2	Diagnostic

(74)	Melanoma	SDS-PAGE	Literature	1	SID-SRM	Tissue	–	192	BIM	1	Therapeutic

(75)	Melanoma	SDS-PAGE	Literature	82	SRM	Tissue	–	–	HSP70; VEGFR2; mTOR; IRS-4; GSK3; AKT1/2	6	Therapeutic

(76)	Melanoma	–	Literature	1	SID-SRM	Tissue	0	10	SNCA	1	Prognostic

(77)	Mesothelial	Glycopeptides enrichment	MS-CSC; spectral libraries	36	LF-SRM	Serum	26	49	Intercellular adhesion molecule 1; basement membrane-specific heparan sulfate proteoglycan core protein; anthrax toxin receptor 1; serum paraoxonase/arylesterase 1; hypoxia upregulated protein 1; thrombospondin-1	6	Diagnostic

(78)	Oral	–	Literature	14	LRP-SRM	Saliva	8	22	C1R; LCN2; SLPI; FAM49B; TAGLN2; CFB; C3; C4B; LRG1; SERPINA1	10	Prognostic

(79)	Ovary	Combinatorial peptide libraries 2D-LC	LF shotgun proteomics	134	LF-SRM	Ascites fluid	–	6	AMBP, BCAM, BTD, CD109, CD14, COMP, CPN2, ECM1, FSTL1, HABP2, HSPG2, IGFBP3, KLK6, LBP, LGALS3BP, MIF, MSLN, MSN, MSRA5, PON1, PRG4, SERPINA10, SERPINC1, SERPIND1, SERPINF1, SHBG, TGFB1, THBS1, TIMP1, TNC	30	Diagnostic

(11)	Ovary	Affinity depletion of 12 abundant proteins; MudPIT; OFFGEL	LF shotgun proteomics	51	SID-SRM	Ascites fluid	25	5	GAPDH, MSLN, PKM1/2	3	Diagnostic

(80)	Ovary	Affinity depletion of 14 abundant proteins	LF shotgun proteomics	2	SID-SRM	Tissue	10	11	WDR1	1	Therapeutic

(81)	Ovary	Affinity depletion of 12 abundant proteins	Literature; computational prediction	34	SID-SRM	Plasma	68	16	AACT, APOA1, APOE, B2MG, C1R, CFAB, CO5, CO6, CO7, GELS, HPT, IC1, ITIH4, RET4, SHBG, TETN, THBG, TRFE, TTHY	19	Diagnostic

(82)	Ovary	Affinity depletion of 20 abundant proteins; SDS-PAGE	LF shotgun proteomics	7	LF-SRM	Plasma	15	18		3	Diagnostic

(83)	Ovary	Affinity depletion of 20 abundant proteins; SDS-PAGE	LF shotgun proteomics	2	LF-SRM	Plasma	1	3	CLIC; TPM	2	Diagnostic

(84)	Pancreas	SDS-PAGE	Literature	1	SID-SRM	Cyst fluid	5	10	KRAS	1	Diagnostic

(85)	Pancreas	–	LF shotgun proteomics	18	LRP-SRM	Tissue	–	9	TGM2, PSAP, DPYSL3, SERPINF1, ARPC4, BRRP7, S100A11, CAN2, MVP, GC	10	Prognostic

(86)	Pancreas	2D-nano-HPLC	iTRAQ shotgun proteomics	1	SRM	Tissue	3	7	Dihydropyrimidinase-like 3	1	Therapeutic

(87)	Pancreas	Affinity depletion of six abundant proteins	Literature; database	260	SID-SRM	Plasma	100	84	KLKB1, IGFBP2, THBS1, PPBP, TXN, LDHB, IGFBP3, LRG1, C5, AGT, CPN2	11	Diagnostic

(88)	Pancreas	Lectin affinity chromatography	TMT shotgun proteomics	1	SID-SRM	Serum	115	26	Serotransferrin	1	Diagnostic

(89)	Pancreas	Membrane and cytosolic fractions	Literature	25	SID-SRM	Tissue	–	10	dCK	8	Treatment

(90)	Pancreas	Affinity depletion of albumin and IgG	Shotgun proteomics (literature)	5	SID-SRM	Plasma	40	20	Gelsolin, lumican, TIMP1	3	Diagnostic

(91)	Pancreas	Laser microdissection; immunohistochemistry	Shotgun proteomics	170	LRP-SRM	Tissue	5	8	ECH1, GLUT1 (GTR1), OLFM4, STML2	4	Prognostic

(92)	Pancreas	Streptavidin affinity 2D-nano-HPLC	Shotgun proteomics	4	SRM	Tissue	38	62	FN1, PRELP, TGM2, AGRN	4	Diagnostic; therapeutic

(93)	Pancreas	Affinity depletion of high and medium abundant proteins	SILAC shotgun proteomics	72	SID-SRM	Serum	20	20	cystatin M, IGF binding protein 7, villin 2	3	Diagnostic

(94)	Pancreas	–	Shotgun proteomics PTM (literature)	1	SID-SRM	Plasma	21	70	α-fibrinogen containing Hyp-530 and Hyp-565	1	Diagnostic

(95)	Prostate	–	Databases, LF shotgun proteomics	32	LF-parallel reaction monitoring (PRM)	Urine	15	15	PROS1; HPR; PZP; SLAIN1	4	Diagnostic

(96)	Prostate	SCX	Literature	1	SID-SRM	Serum	–	–	PSA	1	Diagnostic

(97)	Prostate	Depletion of 14 abundant proteins; immunoprecipitation	Literature	2	SID-SRM	Serum	–	–	proPSA; PSA	2	Diagnostic

(98)	Prostate	Glycopeptides enrichment	LF shotgun glycoproteomics	39	SID-SRM	Serum; Tissue	66	77	GALNTL4; FN; AZGP1; BGN; ECM1		Diagnostic; Prognostic

(99)	Prostate	Affinity depletion of abundant proteins; MCX	Literature	1	SID-SRM	Serum	4	5	PSA		Diagnostic

(100)	Prostate	–	2D-DIGE-MS		SID-SRM	Urine	14	11	Vinculin; PAP; galectin-3	3	Diagnostic; prognostic

(101)	Prostate	Microdissection; glycopeptides enrichment	LF shotgun glycoproteomics	548	SWATH	Tissue	10	65	*N*-acylethanolamine acid amidase; protein tyrosine kinase 7	3	Diagnostic; prognostic

(102)	Prostate	High PH RP-HPLC	Literature	1	SID-SRM	Serum	1	2	PSA	1	Diagnostic

(103)	Prostate	Affinity depletion of 14 abundant proteins; high PH RP-HPLC	Literature	2	SID-SRM	Urine; serum	23	14	ARG2; PSA	2	Diagnostic

(104)	Prostate	Glycoprotein enrichment	Literature	37	SID-SRM	Serum	0	37	CPM; APOB; CADM1; CFH; CP; CTSD; GOLM1; TIMP1	8	Therapeutic

(105)	Prostate	Affinity depletion of 7 abundant proteins	Literature	10	SID-SRM	Seminal liquid; blood plasma	–	37	SNP-L132I	1	Prognostic

(106)	Thyroid	–	Literature	21	LF-SRM; SID-SRM	Tissue	9	27	S100A6; S100A11; ANXA1; S100A13; S100A4; S100A10; ANXA2	7	Diagnostic; prognostic; therapeutic

*A variety of different targeted proteomic methods were employed and are also described*.

**Table 2:** **A compilation of some of the most relevant studies that made use of in vitro, xenografts, or samples from non-human models for the search of molecular biomarkers relevant to the diagnosis, prognosis, and/or therapeutical strategies in cancer research**.

Reference	Type of cancer	Methodology				Sample	Species	Statistically validated targets		Type of biomarker
		Prefractioning/enrichment	Target selection	Number of targets	Mass spectrometry (MS) approach			Protein ID	Number of proteins	
(107)	Breast	IMAC (phospho)	SILAC shotgun proteomics	100	Parallel reaction monitoring (PRM)	MCF7, PC3, HL60 cells	*H. sapiens*	MAPK, PI3K/mTOR, and CDK	–	Therapeutic

(108)	Breast	–	Label-free (LF) shotgun proteomics	258	LF-SRM	MCF10A cells	*H. sapiens*	CDH1; CDH2; vimentin		Therapeutic

(109)	Breast	Phosphatase treatment	Literature	4	Labeled reference peptide (LRP)-SRM	MCF-7 cells	*H. sapiens*	ER, HER2, RAF, and ERK1	4	Prognostic

(110)	Breast	SCX	Literature	76	Stable isotope dilution-selected reaction monitoring (SID-SRM)	MCF-7 cells	*H. sapiens*	SLC2A1, HSPA5, LDHA, PGR, and TFF1	12	Prognostic

(111)	Breast	–	SILAC shotgun proteomics	8	LF-PRM	231-BR; MDA-MB-231 cells	*H. sapiens*	MMP1; EFNB1; STOM1; UAP1; MYCT1; TGM2; S100A4; LCP1	8	Prognostic

(112)	Breast	Avidin-agarose	Isotope-coded ATP-affinity shotgun proteomics	120	SID-SRM; isotope-coded ATP-affinity	MCF-7 cells	*H. sapiens*	CHK1, CDK1, and CDK2	120	Therapeutic

(113)	Breast	Immunoenrichment; SDS-PAGE	Literature	1	SWATH and MS1 filtering	SK-BR3 cells	*H. sapiens*	ErbB2	1	Diagnostic

(114)	Breast	Immunoprecipitation of decorin and periostin	Isobaric tags for relative and absolute quantitation (iTRAQ) shotgun proteomics	2	SID-SRM	MDA-MB-231; T47D; BT-20 cells	*H. sapiens*	Decorin; periostin	2	Therapeutic

(26)	Breast	2D-LC	LF shotgun proteomics	319	SID-SRM	30 cell lines	*H. sapiens*	CLTC, DPYSL2; ABAT; many others		Diagnostic; Prognostic

(115)	Breast	2D-gel	2D-DIGE	11	LF-SRM	ZR-75-1, MDA-MB-231, and MCF-10A cells	*H. sapiens*	HSP105, KRT8, KRT18, RPLP0, and RAD23B	5	Therapeutic

(116)	Breast	2D-LC	iTRAQ shotgun proteomics	12	SID-SRM	MCF7 cells	*H. sapiens*	HSPA8, LDHA, NACA, CTSD, PKM2, IGF-1R	6	Therapeutic

(29)	Breast	–	Transcriptomics database; LF shotgun proteomics;	20	SID-SRM	SK-BR-3 and MDA-MB-231 cells	*H. sapiens*	KPNA2; CDK1	2	Prognostic

(117)	Breast	Cell nuclei enrichment	Literature	2	LF-SRM	MCF-7 cells	*H. sapiens*	NF-κB2; Stat1	2	Prognostic

(118)	Breast	–	LF shotgun proteomics	60	LF-SRM	Tissue	*Mus musculus*	Osteopontin and fibulin-2	2	Diagnostic; Prognostic

(119)	Colon	Lectin affinity	Literature	2	LRP-SRM	WiDr cells	*H. sapiens*	TIMP1; PTPK	2	Diagnostic

(42)	Colon	Laser microdissection; SDS-PAGE	Literature	22	SID-SRM	HCT116, HT29, KM12, SW620, KM12C, KM12L4A, and KM12SM cells	*H. sapiens*	B-catenin; c-Src; c-Myc; PP2A	4	Prognostic

(120)	Colon	Affinity depletion of 3 abundant proteins; SCX	SILAC shotgun proteomics	9	SID-SRM	Serum	*M. musculus*	MGAM; COL1A1; ITIH3 and F5	4	Diagnostic

(121)	Colon	affinity depletion of 3 abundant proteins; SCX	SILAC shotgun proteomics	20	SID-SRM	Serum	*M. musculus*	Cysttin C, secreted phosphoprotein 1, pyruvate kinase 3, procollagen C-proteinase enhancer, nucleobindin, HSP1A, nucleolin, fibronectin, profilin, HSP8	10	Diagnostic

(122)	Colon; lung; melanoma; leukemia; myeloma	SDS-PAGE	Literature	221	SID-SRM	Multiple cell lines (>25)	*H. sapiens*		95	Therapeutic

(123)	Glioblastoma	Secretome enrichment	Shotgun	65	SID-SRM	U87 cells	*H. sapiens*	EGFR, EGFRvIII, and/or PTEN	62	Therapeutic

(124)	Kidney	Immunoaffinity	Literature	1	SID-SRM	PRC3 cells	*H. sapiens*	CA12	1	Diagnostic

(125)	Kidney; Breast	–	Shotgun	114	LRP-PRM	BT474 and Sum159 xenograft	*H. sapiens*	HER2	104	Diagnostic

(126)	Leukemia	IMAC (phospho)	LF shotgun proteomics	25	LF-SRM	AML-193, CMK, CTS, HEL, Kasumi-1, KG-1, MV4-11, and P31/FUJ cells	*H. sapiens*	PI3K, MEK, and JAK	3	Therapeutic

(127)	Lung	SDS-PAGE	Literature	1	LF-SRM	A431 cells	*H. sapiens*	6 phosphosites in EGFR	1	Therapeutic

(63)	Lung	Enrichment for desthiobiotinylated and tyrosine-phosphorylated peptides	LF shotgun proteomics	264	LRP-SRM	HCC366 and H2286 cells	*H. sapiens*	Kinases		Therapeutic

(64)	Lung	Microdissection	Literature	1	SID-SRM	Xenograft cell culture	*M. musculus*	EGFR	1	Therapeutic

(128)	Lung; skin; colon	Phosphotyrosine immunoenrichment	Literature	83	LRP-PRM	A431; SW480 and 11–18 cells	*H. sapiens*	EGFR; FLK2; EPHA1; FAK1; FGFR2; IGF1R; LYN; PGFRA; PTK7; SRC; VGFR2; and YES	21–28	Therapeutic

(129)	Multiple myeloma	SDS-PAGE	Literature	15	SID-SRM	RPMI-8226; U266 cells	*H. sapiens*	NF-κB1 and 2, RelB/p50, Bcl-2, Mcl-1, Bfl-1, Bcl-xL, Bid, Bim, FANCD2, FANCI	–	Therapeutic

(130)	Ovary	Affinity depletion of 20 abundant proteins; IEF; SDS-PAGE	LF shotgun proteomics	14	LF-SRM	Xenograft mouse models	*M. musculus*	AGRN, PSME2, TPI1, DDAH2, GM2A, YWHAB, YWHAH, PSMA, PSMB1–4		Diagnostic

(131)	Ovary	–	Literature	1	Ion-trap LRP-pSRM	2008 cell line	*H. sapiens*	SOD1	1	Therapeutic

(132)	Ovary	Immunoprecipitation; SDS-PAGE	Shotgun	2	SID-SRM	OVTOKO, OVISE, MCAS, OVKATE, OVSAHO, OVMANA, OVSAYO, OVCAR-3, RMG-I, and RMG-II cells	*H. sapiens*	Brg1; ARID1A	2	Diagnostic

(84)	Pancreas	SDS-PAGE	Literature	1	SID-SRM	DLD1; COLO-205; SW480; A549; HCT116; HT-29 cells	*H. sapiens*	KRAS	1	Diagnostic

(89)	Pancreas	Membrane and cytosolic fractions—centrifugation	Literature	25	SID-SRM	PK9, CFPac-1, PK1, SUIT2, AsPC1 human cells	*H. sapiens*	dCK; UMP-CMP; cN-III; ENT1	4	Therapeutic

(133)	Prostate	–	Literature	1	SID-SRM	VCaP and LNCaP cells	*H. sapiens*	ERG3	1	Diagnostic; prognostic

*A variety of different targeted proteomic methods were employed and are also described*.

## Proteomic Studies and the Timely Shift from Classic to Modern Strategies

### Shotgun Proteomics

Within MS-based proteomics, discovery-based experiments, also known as shotgun proteomics, are still the most widely used approaches and can be further categorized into two major groups: label-based technologies (using isotopic or isobaric tags) and label-free (LF) MS-based proteomics (134). Discovery strategies are frequently used to obtain a broad overview of the proteins in a comparative analysis of different cellular characteristics in cancer, such as distinct invasiveness and proliferation, and it has been previously used as a valid tool for high-throughput biomarker discovery in which efficient clinical validation may or may not be achieved (135).

Classic, or shotgun, proteomic tools and strategies, albeit highly efficient in terms of sorting out hundreds or even thousands of proteins involved in any given number of biological states and possibly comparing separate experimental conditions, lack an intuitive or direct connection to steps involving hypotheses formulation, since it seldom involves narrower questions regarding the changes undergone by specific molecules within the different conditions studied. Rather, traditional proteomic experimental designs are conceived in order to come up with complex answers to somewhat non-specific questions, i.e., identifying a huge number of proteins that might be up- or downregulated in different experimental conditions. While this general design has allowed ongoing advances in a number of medical subjects, it usually falls short of providing clinically relevant, practice-changing information and more often than not, fails to provide promptly available tools for better diagnosis or treatment of diseases. This is at least in part due to the fact that the extremely complex results of shotgun proteomic studies bear an inherent gap toward the more specific questions that might be of use for creating novel diagnostic steps or therapeutical strategies.

In common workflows for shotgun proteomics, proteins (either labeled or not labeled with stable isotopes) are enzymatically digested and the resulting peptides are separated by single or multi-dimensional chromatography to be later injected into a tandem mass spectrometer for ionization and analysis. Inside the mass spectrometer, the ionized peptides are driven by electromagnetic fields and analyzed according to their mass-to-charge ratio (*m*/*z*) and precursor ions can then be selected for fragmentation by different MS/MS methods. The ionized fragmented peptides undergo another step in order to have their *m/z* determined. After the steps above, thousands of MS and MS/MS spectra are obtained and can provide information about the amino acid sequences of the peptides and the relative (or in some cases, absolute) abundance of the respective proteins in the original sample (20, 136, 137).

Although this approach promotes the identification of thousands of proteins in a single experiment with high accuracy and resolution (11), it holds grave limitations in real-life scenarios. One major caveat of shotgun proteomics is the fact that digested and fragmented peptides are usually represented relative to the abundance of each protein in the sample, causing the most abundant proteins to be more likely identified, while possibly overlooking less abundant proteins (138). The stochastic nature of precursor ion selection also represents a serious limitation of discovery-based proteomics since it limits the reproducibility of these types of assays. A final relevant obstacle for the propagation of these strategies in biomolecular studies is the fact that classic proteomic studies are technically challenging, limiting the number of laboratories that can cope with its many technical difficulties (9).

The unique technical limitations and strengths of shotgun or discovery-based proteomics have established an unforeseen stalemate: an increasing number of basic-science laboratories have been applying powerful MS tools in a perpetual cycle of proteomic identification, which does not serve a particularly clinically applicable cause, usually not culminating in proposing novel surrogate endpoints; on the other hand, laboratories involved in more hypothesis-driven and clinically applied research tend to rely in less accurate protein identification/quantification methods, such as western blotting and other antibody-based assays (9, 139). To further corroborate the current state, it has been demonstrated that the bulk of papers reporting findings in specific human proteins are focused on a relatively or disproportionately small number of molecules and that the advent of genome sequencing and the resulting growth in proteomic techniques has not changed this imbalance in a relevant fashion (139), making it clear that the mere identification of a humongous number of proteins present in different biological conditions is not enough to provide potentially clinically applicable results. Rather, a more targeted, hypothesis-driven approach, that takes into consideration known biological data should at some point work alongside this broader way of looking at the proteome.

According to the Merriam-Webster dictionary, the scientific method is defined by *principles and procedures for the systematic pursuit of knowledge involving the recognition and formulation of a problem, the collection of data through observation and experiment, and the formulation and testing of hypotheses*. This relationship between specific hypothesis postulation and its testing through experimentation is clearly more in sync with the aspects that drive targeted proteomics. It is important, however, to be clear that discovery proteomic methods are not by any means irrelevant or wasteful in nature. Such strategies have paved the way through which modern targeted proteomics travels and it could be argued that the difficulty in transitioning to more elegant platform for proteomic exploration from broader, discovery-based approaches into hypothesis-driven scenarios in cancer research represents a natural and predictable growing pain in balancing the inherent opportunity costs of each methodology. Shotgun approaches have indeed opened up the proteome for more specific, reproducible, and question-based investigation by targeted proteomics that may allow us to answer more specific questions more rapidly and more accurately than previously possible, in a way that impacts diagnosis and treatment of a number of diseases, including cancer, since targets studies are by definition better for streamlining biomarker discovery in real-life conditions. In reality, the present review demonstrates that this is an ongoing trend that has already helped with the identification of several biomarkers for a number of cancer types, regarding several relevant aspects of the disease, such as diagnosis, staging, prognosis, and therapy.

### Targeted Proteomics

The concept of targeted MS assays for peptide quantitation originated with the study of isotopically labeled peptides used as internal standards to measure peptide levels in biological samples as early as in 1983 (140). Later, in 1990, Kusmierz et al. (141) applied the acronym MRM (multiple reaction monitoring) for the quantification of peptides in human tissue extracts by comparing spectra of unlabeled samples to those of labeled peptides. Modern targeted approaches, generally called selected reaction monitoring (SRM, which can be interchangeably termed MRM) focus on following up the quantification of a handful of proteins of interest in separate experimental conditions, performing relative or absolute quantification with high specificity and sensitivity (46), even in particularly complex backgrounds.

To identify proteins in a targeted fashion, it is mandatory to monitor proteotypic peptides (PTPs), which are unique amino acid sequences that consistently identify a specific protein in a given proteome interrogated by MS (95). These PTPs serve as a signature for the selected protein of interest and are monitored throughout the experimental run. The selection of peptides, which best represent a protein of interest, is a crucial step in the analysis of the sample (20, 142, 143), since targeted proteomics is no more than a method for monitoring the precursor and fragment ions of previously selected peptides, meaning that the proteins under investigation must be known beforehand.

Targeted approaches are driven by specific hypothesis, so previous in-depth knowledge about the protein of interest is necessary. Information used to select candidate peptides to be monitored for targeted approaches can be of varying sources, making the use of previous findings in the literature or even introductory overall screening discovery-based proteomic assays possibly essential for reliable results (144). In the conventional method for SRM, all the information about the target peptides and parameters for their best separation, ionization, and fragmentation must be set prior to the experiment. Such strategy requires access to the host of information about the target proteins, since a good selection of PTPs and peptide fragments ensures high sensitivity and specificity, as mentioned before.

The advent of targeted techniques mitigated the problem around quantification of low-abundance proteins inherent to shotgun methodology, while allowing better quantitative representation of proteins of interest, thus representing a more sensitive and efficient technique as compared to conventional methods for the analysis of specific proteins (47, 144). The targeted approach is a directed and selective analysis, which allows it to function as a form of qualitative and quantitative validation.

Other technical advantages of the targeted approach are related to the selectivity and dynamic range, with the possibility of it being benefited from shotgun strategies in terms of data density and effectiveness (145). Targeted assays can also add multiplexing capabilities that far exceed those of immunological assays, though yet not comparable to discovery-based strategies. Usually the approaches regarding targeted proteomics are precise in quantitative measurements at the peptide level.

A classical targeted proteomics assay requires specific MS instruments like the triple quadrupole analyzer, where the first and third quadrupoles work as *m/z* filters, while the second quadrupole acts as a collision cell (146). The first quadrupole selects the previously defined PTPs based on their *m/z* ratio and the second quadrupole acts as a collision chamber, fragmenting the selected peptides. The precursor and product ion pairs are often called transitions. These fragments (product ions) are analyzed in the third quadrupole where they might also be further filtered for specific fragments to be monitored (146, 147).

**Figure 1:**
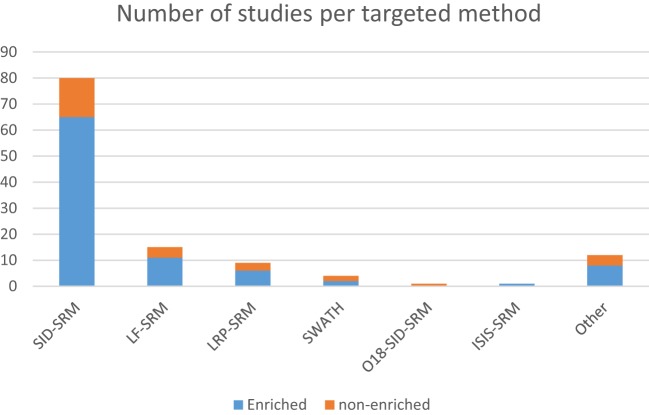
**Distribution of the number of studies using targeted approaches according to the methods used, further grouped by the use of sample enrichment prior to RP-LC-MS**.

Important concepts related to more advanced experimental strategies involve the relative and absolute quantification of proteins that can be achieved using targeted proteomics approaches. Relative quantification (also referred as differential expression) experiments provide the abundance ratio of proteins comparing two separate conditions, while absolute quantification offers the absolute molar (or mass) concentration of specific proteins in the analyte. In order to perform absolute quantification assays, synthetic peptides are spiked into the samples at known amounts for later comparison of the precursor and fragment peak areas of the same peptide between the naturally occurring molecule in the sample and the spiked synthetic one (148). This kind of method has been successfully used in cancer-targeted research by the majority of the authors in the present review, with few variations to the general method. Absolute quantification methods using synthetic labeled peptides are often used for validating biomarkers by targeted proteomics (143). The stable isotope-labeled/stable isotope dilution (SIL/SID) methods represent the gold standard for rigorous proteins quantification through SRM in which peptides are synthesized with the insertion of stable isotope labels at the C-terminal Arg and Lys residues (^13^C_6_^15^N_4_ for Arg, ^13^C_6_^15^N_2_ for Lys) (65, 73, 128). The most commonly used approach relies on isotopically labeled reference peptides (LRPs) that are chemically identical to the light native peptides (AQUA peptides) (149). The analytical precision of this method is high and can result in as low as 5% errors in the estimation of the amount of peptide originally loaded onto the liquid chromatography system (150), but limitations such as cost make its application in broad multicandidate studies impractical. LRP in sample internal standard and LF quantification methods represent alternative tools for protein quantification in SRM (Figure 1), where, in case of the former and the latter, a single peptide (isotopically labeled or not) is used as the reference peptide for all other peptide analytes in the sample; and where, in case of the lattermost, no internal isotope is employed at all, so that quantification is based only on the peak areas extracted from the SRM product ion chromatograms (68, 73).

Another perhaps less-known targeted approach can make use of hybrid instruments such as quadrupole–time of flight that measures the *m/z* of the ions produced using the time of flight of the peptide inside a chamber under vacuum. Hybrid instruments as the QExactive™ (Thermo Scientific) appeared in the targeted proteomics scene to improve the resolving power and speed of targeted analysis (65, 95). Parallel reaction monitoring (PRM) is an excellent choice for the monitoring of low-abundance peptides in biological samples. The PRM acquisition simultaneously analyzes all fragment products generated by peptide fragmentation. This method demands much less investment in optimizing the acquisition schedules, because of its tolerance to coelutions between product ions. A more thorough description of the improvements to targeted proteomics that were brought about by PRM analysis can be found in Ref. (147). Both SRM and PRM hold an intrinsic limitation as to the fact that once the data for the set of peptides specified for the assay is acquired, there is no possibility of revisiting the selection parameters to perhaps look at the data focusing on different targets. This can at least in part be ameliorated through the use of data-independent acquisition (DIA) methods, more specifically by the use of SWATH-MS (sequential window acquisition of all theoretical fragment ion), where sequential windowed acquisition of all theoretical ion spectra within a user-defined retention time and mass-to-charge ratio interval is achieved, so that the coverage of proteins to be monitored in a single assay is greatly expanded, while maintaining the advantageous quantitative aspects of the target acquisition (48, 151). The numerous fragmented ions contained in a map created during this type of MS method represent a gap-free or continuous data set that can be used for sequential *in silico* hypothesis testing, even in cross study comparisons not envisaged in the primary studies (48). Moreover, the inherent properties of the DIA-based aspect of SWATH-MS allow for indefinite remining of the fragment ion spectra using classic target analysis strategies (152), which theoretically allow for increased robustness in biomarker discovery, as shown in a later part of the present review.

The association of different immunoenrichment methods alongside SRM has been used in cancer biomarker discovery in testicular, prostate, and lung tumors (153). Accurate validated MS methods are essential for the application of targeted proteomics in any personalized clinical decisions as reviewed by Abbatiello and colleagues (154). High-resolution instruments can provide increased selectivity of targeted analysis over SRM and allow for the simultaneous execution of both discovery and verification phases and accurately provide mass measurements, which translates into improved selectivity by discriminating more efficiently the targeted peptides from the interferences of complex biological matrices (155).

The National Cancer Institute (USA) started the Clinical Proteomic Tumor Analysis Consortium (156), tasked with evaluating targeted and discovery technologies for quantitative analysis in tissues and biofluids in order to identify proteins that are related to alterations consequent to cancer, serving as a public resource that facilitate access to well-established assays and accelerates the standardization of the technology. Several specialized software, such as Skyline and ATAQS, have been developed to support the application of SRM assays and optimize data analysis over the years (157, 158).

Important large-scale studies have demonstrated the high reproducibility of multiplexed SRM assays performed across multiple laboratories and instrument platforms. With standardized protocols for sample preparation, data acquisition, and analysis, intra- and interlaboratory coefficients of variation in the range of 10−25% were achieved. LoD (limit of detection) and LoQ (limit of quantification) values observed in unfractionated plasma were in the high hundreds of nanograms per milliliter to low micrograms per milliliter concentration ranges for target proteins and had a linear dynamic range spanning three orders of magnitude (22).

Below, we describe several applications where targeted proteomics have in one way or another provided relevant information for the advancement of cancer research, ranging from novel biomarker discovery, all the way to patient stratification, disease prognosis, and treatment. It is important however to stress that methods involving antibody selection of proteins, such as reverse-phase protein arrays (RPPA), were not within the proposed scope of our discussion.

## Targeted Proteomics in Cancer

As shown above, targeted proteomics generates data that complement and enhance pathology diagnosis in cancer. Variations in protein concentration provide information that connects genotype to phenotype. These type of data are of pivotal importance in understanding biological processes in complex diseases such as cancer, which involves the orchestrated interactions of multiple genes and proteins (159).

The most clinically relevant and therefore ideal output from proteomics research is obviously the reliable identification and quantification of cancer surrogate endpoints (i.e., biomarkers validated to predict clinical outcome in an accurate and reproducible manner) in easily accessible patient biofluids, such as plasma, serum, saliva, tears, urine, or feces (160, 161). In reality, different kinds of molecular targets have been used to discover biomarkers in blood/plasma/serum, body fluids, tissues, and their secretions (162). Plasma is a particularly attractive sample source for disease biomarker discovery and early disease prediction, but characterizing the proteome of biological fluids presents daunting challenges due to the extreme complexity and large dynamic range in protein concentrations inherent to these types of samples (162). The choice of plasma or serum as a base sample is an important consideration when developing blood-based biomarker assays, since the expression or availability of individual proteins for processing may vary greatly between these media. This conundrum becomes evident when one acknowledges the lack of consensus among protein biomarker studies (10). Additionally, inter- and intratumoral heterogeneity observed in cancer studies represent yet another obstacle for the discovery and validation of clinically relevant biomarkers.

Targeted approaches are specifically promising for this endeavor due to the possibility of accurate protein quantification and validation in a large number of complex samples, where high-sensitivity MS-based targeted proteomics can be used to overcome some of the aforementioned obstacles.

Some successful applications of SRM/MRM-based proteomics on a variety of biological samples from clinical specimens have been achieved. Tissue-based targeted proteomics applied to cancer plays an important role in disease staging and represents a potential source of candidate biomarkers for early cancer diagnosis, while also playing an important role in enabling large retrospective biomarker discovery and verification. Nevertheless, it suffers from a number of drawbacks regarding reproducibility, scalability, and feasibility of mining formalin-fixed paraffin-embedded (FFPE) biobanks for candidate biomarkers (125). In addition, targeted MS workflows applied to FFPE samples are complementary to techniques requiring high-quality antibodies, such as IHC or RPPA. As for IHC and RPPA, SRM relies on the measurement of the target protein by measuring one or ideally several surrogate peptides (143).

Chen et al. accomplished a quantitative SRM protein analysis of urine samples, resulting in 12 proteins with higher concentration in bladder cancer patients when compared to control samples (21) in a pioneering study in 2012. Multiplexed SRM analysis generated a six-peptide marker panel involving adiponectin, afamin, apolipoprotein A-II precursor, complement C4 gamma chain, and prothrombin, all of them theoretically able to discriminate bladder cancer patients from non-cancerous individuals.

In addition, enrichment of target proteins before SRM has proven to be useful for reliable quantification (Figure 1) in the low microgram per liter range, with several studies showing LoQs below the microgram per liter range, even in relatively complex backgrounds. In this context, immunoextraction with antibodies immobilized on a hydrazide resin, followed by digestion of the immunoprecipitated proteins with tandem isotopic-labeled SRM assays, has resulted in low microgram per liter quantification of proteins such as carcinoembryonic antigen in lung cancer through blood serum samples and found spiked levels of other proteins such as tissue factor pathway inhibitor 2 and secretory leukocyte peptidase inhibitor (67).

Although many candidate molecules have been proposed over the hundreds of studies published, only 23 protein plasma biomarkers have been cleared by the US Food and Drug Administration (FDA) since 2003 as clinical biomarkers averaging less than two proteins per year over the last 12 years (163). Among the FDA-approved or -cleared cancer biomarker proteins, nine are exclusively designed for serum and six others are applicable to either plasma or serum. Recent SRM studies for cancer biomarker verification show a similar trend regarding the proportion of potential biomarkers found in serum and plasma samples (164, 165). Despite recent technical advances, there are still huge analytical challenges for clinically relevant identification of biomarkers in serum or plasma.

**Figure 2:**
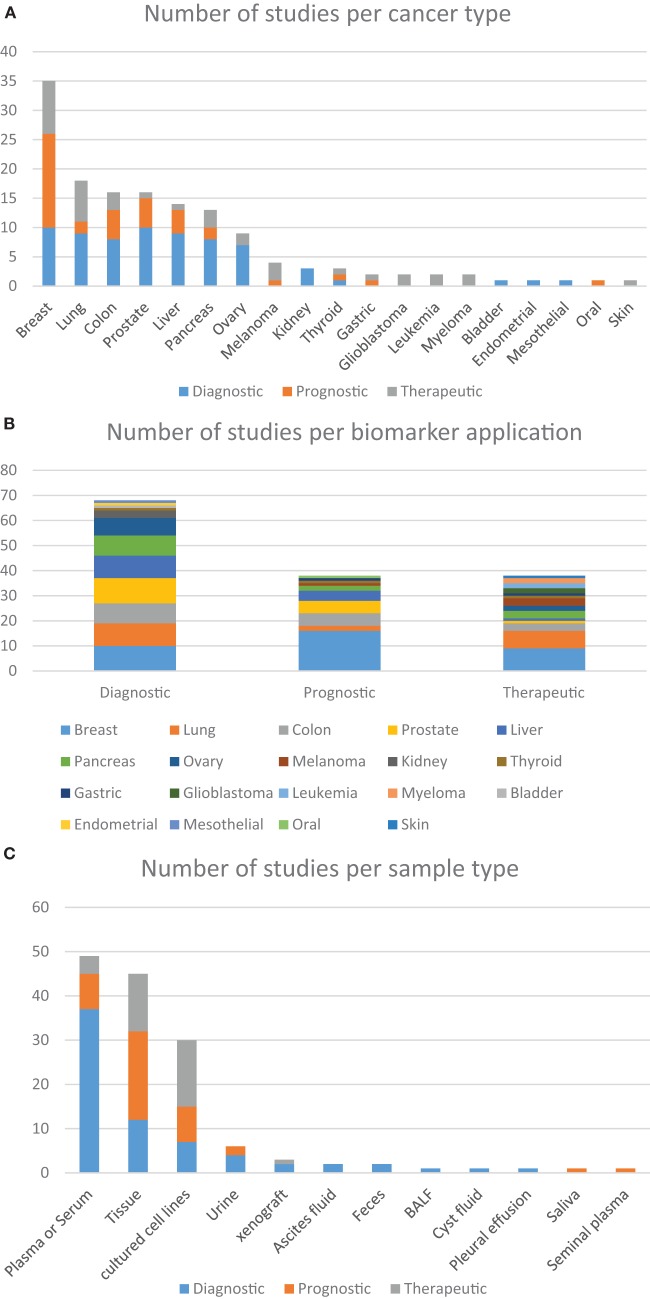
**Distribution of the number of studies according to the approach given by the authors**. **(A)** Based on the use of targeted proteomics dedicated to different types of cancer, further grouped into the clinical application of each biomarker; **(B)** the clinical application of different biomarkers found in within the studied cancer types; **(C)** distribution of the studies using different sample types according to the clinical application of the biomarkers. These charts were based on data from all studies included in this review that focused on the analysis of cancer-related samples, either from human, or from other species or *in vitro* tests.

Studies have previously suggested that whole blood-based targeted proteomic profiling is capable of screening and identifying biomarkers for diagnostic, prognostic, and therapeutic purposes. In truth, several reports can be found in which a host of different sample types are used as alternative sources of data for investigating cancer biology and identifying potential biomarkers, although in-depth quantitative profiling of human plasma samples for biomarkers discovery remains quite challenging. Analyzing the studies selected on Table 1, it is possible to find eight predominant human sample types studied by targeted proteomics, among which, tumor tissue and whole- or fractionated-blood are the most often studied (Figure 2B). Together, these studies demonstrate that robust, reproducible targeted proteomic and subsequent pilot-scale validation studies can be accomplished using LF quantification strategies. Promising results include the findings from Keshishian et al., whom have shown detection limits of target proteins in the range of picograms per milliliter after depletion and sample fractionation of the plasma proteome (150). One lingering challenge of SRM for candidate biomarker verification is the required sensitivity for quantification of low-abundance proteins, given a dynamic concentration range of plasma proteins of over 12 orders of magnitude (166, 167).

It is clear that applying targeted proteomics to the discovery of novel biomarkers with real-life potential of impacting medical practice is a daunting task. The drastic heterogeneity or large biological variations such as gender, age, genetic factors, dietary considerations, environmental factors, and drug treatment often hinder the discovery and establishment of gold standard biomarkers that can be applied to screen the overall population (10). Nevertheless, several research groups have been able to contribute to this effort in a variety of cancer types. Below we describe examples of these advancements regarding particularly relevant types of tumors and the biomarkers that were either proposed or validated during these assays. Upon careful analysis of the data, it is possible to discern interesting trends. Out of the 100-plus studies examined in Tables 1 and 2, most were dedicated to studying biological aspects of breast cancer (Figure 2A). Second, most biomarkers were related to diagnostic applications in favor of prognostic or therapeutical targets (Figure 2B), with the exception seen when the sample source consisted of assays that made use of actual tumorous tissue, where a great dedication toward prognostic biomarkers was evident (Figure 2C). Finally, most of the reports were based in classic SID-SRM quantification, although other less common techniques were also described (Figure 1).

### Breast Cancer

As previously stated, breast cancer is the second most common type of tumor in the human population worldwide, so it stands to reason that great focus has been given to the subject in recent times, with targeted proteomics providing invaluable insights regarding diagnosis, monitoring, and therapy of this kind of cancer (Figure 2A). Narumi et al. applied large-scale phosphoproteome analysis and SRM-based quantitation to develop a strategy for the systematic discovery and validation of biomarkers using tissue samples for breast cancer (28). Through immobilized metal affinity chromatography and isobaric tags for relative and absolute quantitation (iTRAQ) isotopic labeling techniques, they identified phosphopeptides that were differentially abundant between high- and low-risk recurrence groups of breast cancer predicted by MammaPrint, an FDA-approved breast cancer recurrence assay. The identified phosphopeptides were validated by SRM, indeed augmenting MammaPrint prediction capabilities. This systematic approach holds enormous potential for the discovery of bona fide disease biomarkers in cancer research.

Despite the progress made to date in treating breast cancer, the constant changing dynamics of its incidence requires continuous investigation to identify new methods for detection and treatment. In this context, an SRM-based study by Pavlou and collaborators in 2014 suggested that higher levels of 4-aminobutyrate aminotransferase (ABAT) could be related to better prognosis in breast cancer patients, specifically those who are ER positive, tamoxifen treated, and with grade II staging (29). The somewhat small sample cohort (*N* = 20) in addition to the fact that ABAT is yet to be studied in the specific context of breast cancer make it hard to readily export these findings into a clinical scenario.

Shaheed et al. identified proteins whose expression correlate with breast cancer progression and validated candidate profiles of benign, non-invasive, and invasive breast tumors using discovery proteomics, with the use SRM for validation (31). The results corroborated previous reports of higher levels of cofilin-1 and p23 and lower levels of membrane copper amine oxidase (AOC-3) in invasive carcinoma patients when compared to normal controls. In addition, the results have indicated that proteomic analysis of matched tissues can generate a valuable subset of candidate proteins for further investigation.

A handful of studies have focused on analyzing metastatic behavior through targeted proteomics. In one study by Cawthorn et al. the differentially abundant proteins identified in an iTRAQ-2DLC-MS/MS experiment were validated by LF SRM on breast tumor tissue sample set (*N* = 32) (23). Together with tissue microarray analysis data, SRM results indicated the correlation between higher levels of decorin and endoplasmin and the presence of lymph node (LN) and distant metastases.

By using a targeted approach employing MIDAS, a SRM assay was successfully designed based on a limited set of *in silico* predicted transitions in a study aimed at identifying protein level changes in MCF-7 cells in response to temporal IGF-1R stimulation, where ENO1 showed up at higher levels in breast cancer cell versus their normal counterparts; ENO1, PKM2, and LDHA were also upregulated in both invasive MDA-MB-231 cells and in IGF-1-induced MCF-7 cell, which indicated IGF-1 involvement in tumor invasiveness (116).

One study by Greenwood and colleagues in 2012 demonstrated a panel of markers differentially expressed in LN-positive and -negative tumors that enabled further classification into two phenotypes regarding the expression of signal transducer and activator of transcription 1-alpha/beta (STAT1), interferon-induced GTP-binding protein Mx1, and leukocyte surface antigen CD74. Cellular functional assays showed that interferon-induced upregulation of STAT1 was associated with increased cell migration and invasion while CD74 overexpression resulted in increased cell adhesion (168). The authors suggest that targeting the pool of CD74 expressed at the plasma membrane could be a viable strategy for stratified therapy of triple-negative breast tumors overexpressing Stat1 and CD74.

### Colorectal Cancer

Colorectal cancer is the most common gastrointestinal malignancy worldwide and the third leading cause of cancer-related deaths, which makes the discovery and validation of novel biomarker candidates for this type of cancer of the utmost importance for improving disease detection in routine surveillance and screening protocols. Ang and colleagues were able to create a SRM library of PTPs that was applied to the screening of fecal samples from eight CRC patients and seven healthy controls. The abundance of 40 proteins of interest selected from a preliminary discovery-based approach was monitored during the assay (15). Nine proteins were exclusive for the fecal samples of CRC patients, of which only hemoglobin and a-1 antitrypsin had been previously proposed as CRC biomarkers. The remaining seven proteins had not been previously explored as fecal biomarkers. The aforementioned group of nine proteins now represent a novel candidate biomarker panel proposed for the detection of CRC with similar or higher sensitivity than the presently available commercial immunoassays and serve as an option to the standard fecal occult blood screening test which has well-known high false-positive and false-negatives rates. This protein panel, if proven to be validated in a larger cohort of samples, could be reengineered into antibody-based assays, compatible with current clinical analyzers.

### Pancreatic Cancer

The diagnosis of pancreatic ductal adenocarcinoma is particularly challenging since the disease tends to be asymptomatic. Therefore, the investigation of the pancreatic cancer proteome has gained considerable attention over the years. A study by Pan et al. quantified four proteins differentially expressed in the plasma of 20 patients in early stages (I and II) of pancreatic cancer in comparison to 40 controls, half of which had chronic pancreatitis, which shares many clinical symptoms with this type cancer (90). Of the four proteins that were quantified by SRM, tissue inhibitor of metalloproteinase 1 (TIMP1) was also measured by ELISA. The authors suggest that the SRM assay measured total TIMP1, while the ELISA measured only free TIMP1, neglecting molecules bound to self-directed antibodies or other protein complexes.

In this context, Yoneyama et al. examined two proline-hydroxylated sites in alpha-fibrinogen as potential biomarkers for pancreatic cancer (94). Concentration of alpha-fibrinogen remained constant among 27 healthy controls and 70 cancer patients, while concentrations of specific hydroxylated peptides and hydroxylation ratios were both increased in cancer patients (*p* < 0.05). The hydroxylated alpha-fibrinogen peptides had area under the curve (AUC) values of 0.650 and 0.668, respectively, both of which were below the AUC value (0.903) for the FDA-approved pancreatic biomarker, CA19-9.

A couple of studies in 2011 and 2013 reported by Turtoi et al. and Takadate et al. made use of shotgun LF quantification of pancreatic cancer tissues with poor and better survival outcomes, and non-cancerous pancreatic ducts (91, 92). Both studies selected targets for SRM protein quantification. Among these, 14 proteins were found to be upregulated in pancreatic cancer compared to normal tissue. Patients whose tumors expressed the proteins enoyl-CoA hydratase 1 (ECH1), olfactomedin-4 (OLFM4), stomatin-like protein 2 (STML2), and glucose transporter member 1 (GLUT1 or GTR2) had significantly worse survival rates, suggesting that these proteins may be of prognostic value, and may serve as new therapeutic targets. Kawahara and collaborators reported the use of proteomic profiling through iTRAQ for identifying a number of proteins differentially abundant between cancerous and normal pancreatic duct epithelium, with higher abundance of dihydropyrimidinase-like 3 (DPYSL3) in human pancreatic cancer (86). Given that it has important roles in regulating the motile phenotype of pancreatic cancer cells, DPYSL3 may be a candidate for antimetastasis therapeutic strategies, which may ultimately lead to a reduction in the large number of deaths caused by this devastating disease.

### Hepatocellular Cancer

Hepatocellular carcinoma (HCC) mortality is high because early detection is hampered by the high inaccuracy, cost, and procedural risks of the available tests, which creates an urgent need for minimally invasive, highly specific, and sensitive biomarker candidates that enable early disease detection while therapeutic intervention is still potentially beneficial. Lee et al. targeted non-glycopeptide candidates for identification and validation of molecular markers among glycoproteins in human plasma (55).

Ahn et al. studied plasma samples of hepatocellular cancer patients *via* a lectin-coupled SRM approach based on fucose-specific aleuria aurantia lectin fractionation of glycoproteins prior to SRM (49). The study yielded glycoforms of tissue inhibitor of metalloproteinase 1 (TIMP1) and protein tyrosine phosphatasek (PTPk), which play important roles in invasive and metastatic cancer cells and are known targets of *N*-acetyl glucosaminyl transferase (GnT-V). The results obtained using lectin-based glycoprotein enrichment coupled to SRM shed light on biomarkers capable of distinguishing plasma from HCC patients and non-cancerous controls and that aberrant glycoforms of TIMP1 and PTPk were upregulated in GnT-V-overexpressing cells in comparison with their control counterparts.

Kim et al. measured the total amount of AFP (non-glycopeptide level) and the degree of glycosylated AFP in human serum using an SRM assay (52). This assay proved that the quantification of deglycosylated AFP has much better sensitivity and specificity toward differentiating HCC cancer samples from controls when compared to measurements of total AFP. Another study using an SRM approach focused on the verification of vitronectin and clusterin, confirming their significant downregulation in human hepatocellular cancer serum versus normal control serum (60). Mustafa and colleagues have validated and confirmed reduced levels of ApoA1 in blood samples of HCC patients versus hepatitis C virus-infected patients (57). ApoA1 is known to suppress neutrophil activation and inhibit endothelial expression of adhesion molecules (169) and its downregulation may thus lead to HCC, indicating its role as a possible biomarker for HCC diagnosis, prognosis, and monitoring.

### Prostate Cancer

Biomarkers that offer the possibility of detecting early onset of disease progression or that can differentiate between tumors that remain dormant and those that will eventually develop into aggressive invasive malignancies would provide opportunities for an earlier as well as a more targeted intervention, avoiding unnecessary costly treatments and overall improving survivability.

Geisler et al. determined the amount of prostatic acid phosphatase (PAP), vinculin, and galectin-3 by SRM specific to the urine of control patients, non-relapsing prostate cancer patients, and relapsing prostate cancer patients. PAP was significantly higher in the urine of cancer patients, galectin-3 was verified as a prognostic biomarker candidate and secernin-1 has been validated as a diagnostic tissue biomarker with WB analysis (100).

### Ovarian Cancer

Huttenhain et al. proposed additional sample preparation steps aimed toward reducing sample complexity in order to achieve detection of proteins below the low nanograms per milliliter concentration range with the use of existing MS technologies in the study of ovarian cancer (81). The resulting SRM library was applied to accurately and reproducibly quantify 34 known cancer biomarkers with demonstrated reproducibility between 62 ovarian cancer and 16 benign ovarian tumor patients. Moreover, the authors discovered a long set of cancer-associated proteins, among which 83 and 169 were detected, respectively, in plasma and urine samples. The proteomic data were submitted to the Peptide Atlas SRM Experiment Library, and the SRM assay coordinates were made publicly available for use in future cancer studies. The ever growing size of the data provided by SRM will help close the translational gap present in the validation of potential biomarkers, since it allows for the monitoring or simply identification of candidate biomarkers thought the use of a set of SRM coordinated in different laboratories and instrument platforms.

The FDA has approved human epididymis protein 4 (HE4) as a marker for monitoring recurrence or progression of epithelial ovarian cancer (15). Reliable clinical evidence demonstrates that MS-based proteomics of plasma and serum for the identification of HE4, alone or in combination with CA125, improves accuracy of routine screening. The CA125 blood test was granted FDA clearance for use as a monitoring response test in detecting residual or recurrent epithelial tumors in patients after their first-line therapy has failed (170).

Drabovich and Diamandis have used combinatorial peptide libraries in the discovery phase of their proposed experimental design to target low-abundance proteins with LC-MS/MS data-dependent discovery methods and identified low-abundance proteins in ovarian cancer ascites, which were further verified by SRM (79). Kallikrein 6, metalloproteinase inhibitor 1, macrophage migration inhibitor factor, follistatin, and mesothelin were all present in the samples.

Another study analyzed multiple isoforms of seric chloride intracellular channel protein (CLIC1 and CLIC4) and tropomyosin (TPM1-4) as potential biomarkers for ovarian cancer (82). The CLIC1/CLIC4 ratio varied by more than twofold in some samples, with AUC values for CLIC1 and CLIC4 of 0.86 and 0.79, respectively. The AUC values for TPM were dependent on the particular isoform, with 0.72 for TPM3 and 0.81 for TPM4.

Haslene-Hox et al. performed a complex comparative proteomics assay to find differentially expressed proteins in interstitial fluid from ovarian carcinomas compared to endometrial cancer and to healthy tissues (80). Levels of six candidate proteins (CEACAM5, FREM2, MUC5AC, TFF3, PYCARD, and WDR1) were further monitored in individual tumor lysates from ovarian carcinomas using MIDAS, WB, and/or SRM. The conclusion of the orbitrap assays was that WDR1 protein was significantly more abundant in interstitial fluid from ovarian carcinomas versus controls.

### Lung Cancer

Lung cancer is one of the most common types of cancer and has very poor prognosis and high mortality and so it is naturally a common target for proteomic research. Kim et al. quantified 96 potential non-small-cell lung cancer (NSCLC) biomarkers in NSCLC patient versus plasma from the control group (21, 51, 65, 131). An SRM assay quantified 28 transcriptional factors (TF) across eight lung cancer cell lines, revealing 14 differentially abundant TF. Seventeen proteins with high plasma levels were further verified using SRM in a large sample set and one of these proteins, zyxin, was proposed as a candidate biomarker of early NSCLC diagnosis. The haptoglobin beta-chain (70) and serum amyloid A (SAA) (171) were elsewhere identified as new possible diagnostic markers for lung cancer. Park et al. compared and quantified serum levels of target proteins in patients with and without NSCLS by ESI-MS/MS. They identified significantly higher level of HP-alpha chain in lung cancer patients.

A handful of studies utilizing target proteomics for the discovery of biomarkers involved in lung cancer metastasis have been made public to date. Nishimura et al. used SRM-MS techniques to further validate their introductory discovery proteomics findings in a set of lung adenocarcinoma FFPE tissues, where a panel of stage IA and IIIA specific protein levels was demonstrated (68). The results showed hAG-2 as significantly more abundant in stage IIIA metastatic LN when compared to stage IA primary lesions.

Li and colleagues have presented a 13-protein blood-based categorization tool for differentiation of benign and malignant nodules using SRM (66). These proteins (LRP1, BGH3, COIA1, TETN, TSP1, ALDOA, GRP78, ISLR, FRIL, LG3BP, PRDX1, FIBA, and GSLG1) are likely regulated by a group of transcriptional regulators (nuclear factor erythroid 2-related factor 2, aryl hydrocarbon receptor, myc, and c-FOS) related to lung cancer. Such tool can be used to prevent patients with benign lung nodules from undergoing invasive procedures such as biopsy and surgery.

Sung et al. quantified SAA isoforms SAA1 and SAA2 and demonstrated their elevated levels in lung cancer patient serum when compared to healthy controls, both through SRM and ELISA (71).

### Thyroid Tumors

Martínez-Aguilar et al. used SRM for the profiling of isoform-specific expression of the calcium-binding protein S100 in the three most common tumors of the thyroid gland in comparison with normal thyroid tissues (106). Results from SRM analyses were confirmed by metabolic SILAC labeling and WB analysis and allowed the identification of S100A31 as a novel biomarker candidate for papillary thyroid carcinoma. Additionally, the ability to discriminate between follicular and papillary thyroid tumors though monitoring of S100A6, S100A4, and annexin A1 levels was described.

### Targeted Proteomics and Cancer Response to Therapy

A number of studies have attempted to decipher the therapy or drug-induced proteomic changes in cancer, aiming to improve our understanding of the function of drugs and underlying mechanisms for therapy resistance in cancer. Cancer cells can become resistant to drugs in two major ways: the cancer itself changes through mutation so that the targeted therapy is no longer efficient and/or the tumor cells activate alternative molecular pathways that promote tumor growth in a way not influenced by therapeutical intervention. It is important to stress that in this instance the term *targeted* is more loosely applied, as the same word can be used for different therapeutical strategies that do not necessarily involve targeted proteomics.

In a study from 2014, the discovery of components in resistance pathways resulted in several drug-target candidates that have the potential to be used to overcome resistance to the original medication (75). The authors successfully used SRM-based quantitative proteomics to evaluate drug response and predict resistance mechanisms in melanoma. In this context, XL888 treatment reduced tumor growth drastically showing a significant decrease in the expression of proteins from the PI3K/AKT/mTOR signaling pathway, suggesting that this pathway may be alternatively activated to compensate for BRAF inhibition. Moreover, they monitored the molecular response of two NRAS mutant melanoma cell lines submitted to treatment with a MEK inhibitor, AZD6244. Treatment with AZD6244 only led to marginal cell growth inhibition. The results showed that AZD6244 treatment generated a molecular fingerprint with increased Wnt/β-catenin signaling, NFκB phosphorylation, and upregulation and phosphorylation of PDGFR-β, a receptor tyrosine kinase (RTK).

Paraiso et al. showed another example of the PI3K/AKT pathway being involved in drug resistance (74). By using SRM, they discovered a mechanism by which melanoma cells with BRAF mutation and PTEN loss can escape from BRAF inhibition. Inhibitors of BRAF have been developed and recently approved to treat melanoma harboring activated V600E BRAF mutation that is identified in more than 50% of melanoma cases. A subgroup of patients with BRAF-mutated melanoma showed limited response to these inhibitors due to intrinsic resistance. In this study, SRM-based proteomics helped to identify the pivotal signaling node in the resistance mechanism by efficiently monitoring the expression of a group of selected proteins following drug treatment and consequently showed solid evidence for the development of combination therapy in melanoma treatment.

Zhang et al. performed a comprehensive study to uncover EGFR phosphorylation sites correlating with somatic mutation and/or erlotinib (EGFR inhibitor) sensitivity in lung cancer cell lines (73). Three phosphorylation sites (Y1110, Y1172, and Y1197) connected to erlotinib sensitivity were validated using IP-SRM in lung cancer tissue samples; Y1197 phosphorylated peptides showed good signal, which confirmed that IP-SRM strategy is suitable for EGFR phosphorylation measurement. In a follow-up study, phosphorylation sites of EGFR were also monitored using SRM and their sensitivity to tyrosine kinase inhibitor (gefitinib) in the presence/absence of EGF was investigated.

Held and collaborators used MS1 filtering from shotgun proteomics data and SWATH-MS for quantification of tyrosine kinase ErbB2 from SK-BR-3 cells and its modified forms in breast cancer, reporting reproducibility of workflows with different proteases and indicating quantitative competitiveness of SWATH with SRM methods (113).

De Marchi et al. investigated the dynamics and variability of the protein response to the drug tamoxifen in human breast cancer (25). They identified proteins whose dynamics differed widely between cells, in a way that corresponded to the outcomes of either cell death or survival. Upon assessing peptide abundance differences between patient groups in the primary breast cancer tissue confirmatory dataset, only PDCD4 and CGN peptides were found differentially abundant. This study opens the way to understand molecular responses to drugs in individual cells. In tamoxifen-treated breast cancer patients, high levels of EIF3E were significantly associated with prolonged progression-free survival and with therapy resistance. These efforts resulted in the identification of a putative protein profile that is associated with the type of response to tamoxifen therapy. Umar et al. validated the protein EMMPRIN in an independent patient cohort and confirmed its association with tamoxifen therapy resistance in recurrent breast cancer (34). Using the same quantitative approach, Yang and colleagues applied iTRAQ-MS and SRM techniques to investigate innate resistance to bortezomib, which is a proteasome inhibitor approved for multiple myeloma treatment (37, 172). Differentially expressed proteins identified in the MS analysis were selected for subsequent SRM-MS quantification, from which they successfully identified that myristoylated alanine-rich C-kinase substrate (MARCKS), a substrate of protein kinase C (PKC), is a key mediator of drug resistance. Inhibition of MARCKS activity by a PKC inhibitor enzastaurin or siRNA-mediated knockdown significantly enhanced the sensitivity of resistant myeloma cell lines and primary myeloma samples to therapy. They showed that MARCKS inhibition combined with bortezomib treatment could overcome bortezomib resistance and effectively inhibit tumor growth in a multiple myeloma xenograft model. Their findings provide a new biomarker for the prediction of patients who are likely to have response to bortezomib, meanwhile also developed a rationale for a combination therapy targeting proteasome and MARCKS simultaneously, in order to improve the outcome of patients with refractory multiple myeloma.

Xiang et al. used cell line models of multiple myeloma to investigate drug resistance of melphalan by comparing signaling, apoptosis-regulating, and DNA repair component proteins, finding a nuclear factor-kappaB signature (129). Drug-resistant cell lines exhibit consistent decreases in initiators and sensitizers, when compared with naïve cell lines, indicating that potential for induction of apoptosis may be reduced by decreasing their abundance. This important information could help define the molecular basis for personalized cancer treatment in some patients.

In the context of glioblastoma (GBM), the quest for finding useful biomarkers is vital, once this is the most aggressive primary brain tumor, presenting remarkable neo-angiogenesis capabilities in addition to cellular and molecular heterogeneity. As a means to investigate proteins that are altered by antiangiogenic treatment, thereby providing biomolecular signatures of tumor response in GBM, one study identified marker proteins that are altered during treatment and may serve as a short-term readout of antiangiogenic therapy (47). These included malectin, calnexin, calreticulin, lactate dehydrogenase (LDH), and isocitrate dehydrogenase (IDH). The authors addressed the tumor escape mechanisms to bevacizumab and reported that LDHA was found to be induced by the treatment, while the upregulation of malectin and calnexin had not yet been previously connected to antiangiogenic treatment. The induction of LDHA was not only seen in tumor cells but also in the non-neoplastic host compartment, an observation that was possible due to the species-specific SRM workflow established in said study. Whether the modulation of malectin, calnexin, and calreticulin is a result of hypoxia-induced ER stress in response to bevacizumab remains to be determined. The authors speculate that glycoprotein production and maturation may be affected by antiangiogenic treatment, suggesting that glycoproteins could serve as important response markers. In addition, the establishment of a panel of target proteins modulated upon antiangiogenic therapy may provide the robustness required for biomarker-based patient stratification. By demonstrating that GBM-relevant alterations in the EGFR pathway distinctly change the secreted profile of invasion-promoting proteins as well as the expression and phosphorylation status of intracellular proteins, Sangar et al. investigated the effects of EGFR signaling on the secreted levels of proteins implicated in aggressive invasiveness and proliferation of GBM cells (123). Additionally, levels of intracellular and secreted proteins clearly showed that EGFRvIII carrying cells are functioning under higher oxidative stress. These results confirm that introduction of EGFR signaling proteins influences the expression as well as phosphorylation status of multiple proteins in the EGFR network, resulting in differences in the signal transduction to the nucleus (123).

Alcolea et al. reported (i) the emerging notion that cancer cells are not always dependent on single oncogenes, (ii) that markers of PI3K and MEK pathway activities are poor predictors of response to PI3K and MEK inhibitors, respectively, (iii) that kinase pathways can cooperate to drive cancer cells proliferation in some systems, and (iv) that a combination of inhibitors may be more effective in arresting cancer cell growth when compared to agents that target single kinases (126).

Guo et al. observed a breast cancer MCF7 cell population (MCF + FIR) that could survive after a course of clinical fractionated doses of radiation and showed enhanced radioresistance compared to the wild-type parental MCF7 cells (112). The kinases play pivotal roles in triggering breast cell cycle checkpoints and regulating cell cycle progression. In addition, some of these kinases are also actively engaged in the phosphorylation of mediators in DNA repair pathways. Thus, the above findings of global kinome alterations associated with radioresistance provide new knowledge in understand tumor adaptive radioresistance and offer potential targets to sensitize cancer cells toward radiation therapy and to achieve better remission of cancer.

Kim et al. tried to identify mechanisms of drug resistance involving alterations in protein kinase (PTK) signaling pathways in a lung tumor cell model of acquired resistance to the PTK inhibitor erlotinib (128). PTK expression varies between different types and stages of cancer and alterations in PTK expression is an important mechanism of resistance to targeted cancer therapeutics. These considerations suggest that multiplexed, targeted analysis of PTK expression profiles could be valuable in studying mechanisms of drug susceptibility and resistance.

## Conclusion

Targeted proteomics has evolved into an invaluable tool in cancer research and a viable field for the identification of new and improved biomarkers for the development of better diagnostic and therapeutical strategies. It is a technique that can be applied for the precise, accurate, and sensitive quantification of relevant proteins in a range of clinical materials, such as tissues and other bodily fluids and has enabled wondrous developments in areas such as gene therapy, drug target discovery, and personalized therapy.

The clinical and molecular heterogeneity of cancer currently presents clinicians with difficult problems when choosing adjuvant treatment for individual patients. Nevertheless, a huge number of studies have provided novel candidate biomarkers in a host of cancer types that may serve as more efficient therapeutical or diagnostic targets. The fact that targeted proteomics approaches are generally reproducible across different platforms allows for the synergistic effect that may allow for an ever growing increase in the discovery of clinically relevant biomarkers.

A major obstacle for the use of target biomarkers in clinical practice is the requirement for extremely sensitive and specific candidates, which is currently far from perfect. There is a clear issue regarding sensitivity, which is dealt with by some authors through the use of immunoenrichment steps on sample preparation while others rely on the use of crude samples to avoid loss of low-abundance components. An additional pitfall is that the ability to analyze large sample sets is still lacking. The overall sensitivity and specificity shortcomes of target methodologies can only be surpassed through development of more robust multiplexing capabilities, allowing for multiple markers to be detected at once and for many patients to be screened within a reasonable time frame. SWATH (151), NeuCode (173), and Hyperplexing (44, 174) may not be already inserted in the clinical trials, but are a step toward the right direction in addressing these aspects. Another significant issue is the fact that candidate biomarkers seldom bring satisfactory clinical response. It is necessary to develop optimized workflows in which high-quality results are filtered through and interaction between researchers and physicians is made possible (175).

We firmly believe that such progress in clinical proteomics will pave the way for greater success by accumulating and sharing knowledge and experience for better understanding validation criteria for the regulatory authorization of multiplex MS-based assays. The study of the human proteome and the development of platforms capable of coping with the sheer complexity of the many changes that happen in the proteomic profile of patients at the onset and during progression of cancer has opened a new frontier for the identification of predictive, prognostic, and therapeutic biomarkers with significant translational implications and is quickly becoming an essential tool for the delivery of individualized treatment to cancer patients, not only based on patients genotype but also on phenotype. It is important to note that the molecular characterization and quantification of systemic levels of certain biomarkers could better describe biological processes responsible for instabilities of genomic profiles that in turn result in changes in signaling proteins that ultimately give rise to changing phenotypic behavior (137, 176).

The numerous examples found throughout the text of studies that were able to postulate, identify, and validate a variety of biomarkers relevant to a host of aspects of cancer research through the use of a variety of targeted approaches are a convincing statement toward how the ever-increasing use of targeted proteomics tools for cancer research can be groundbreaking in terms of improving diagnosis and treatment of cancer in real-world, clinically relevant scenarios.

## Author Contributions

WF conceived the idea of the article, participated in reviewing each step during the article preparation, and coordinated the parts from different authors. SF and CM contributed equally to this work. Both wrote most of the article, actively searched, analyzed, and discussed the articles reviewed. MF and AS were responsible for reviewing the proteomics methods and techniques. MC and PF critically reviewed, discussed, and proposed ideas for the article.

## Conflict of Interest Statement

The authors declare that the research was conducted in the absence of any commercial or financial relationships that could be construed as a potential conflict of interest. The handling editor declared a shared affiliation, though no other collaboration, with several of the authors (CM, AS, MF, MC, and WF) and states that the process nevertheless met the standards of a fair and objective review.
